# Suppression of long non‐coding RNA TNRC6C‐AS1 protects against thyroid carcinoma through DNA demethylation of STK4 *via* the Hippo signalling pathway

**DOI:** 10.1111/cpr.12564

**Published:** 2019-04-01

**Authors:** Liu‐Xue Yang, Ji Wu, Man‐Li Guo, Yong Zhang, Shao‐Gang Ma

**Affiliations:** ^1^ Department of Endocrinology and Metabolism The Second Affiliated Hospital of Guilin Medical University Guilin China; ^2^ Department of Thyroid and Breast Surgery Suqian Hospital Affiliated to Xuzhou Medical University Suqian China; ^3^ Department of Thyroid and Breast Surgery Nanjing Drum Tower Hospital Suqian China; ^4^ Department of Endocrinology and Metabolism Suqian People’s Hospital, Nanjing Drum Tower Hospital Suqian China; ^5^ Department of Endocrinology and Metabolism Huai’an Hospital Affiliated to Xuzhou Medical College and Huai’an Second People’s Hospital Huai’an China; ^6^ Department of Endocrinology and Metabolism Suqian First Hospital Suqian China; ^7^ Department of Endocrinology and Metabolism The First Affiliated Hospital of Guangxi University of Chinese Medicine Nanning China

**Keywords:** Hippo signalling pathway, long noncoding RNA, proliferation, serine/threonine kinase 4, thyroid carcinoma, TNRC6C‐AS1

## Abstract

**Objectives:**

Thyroid carcinoma (TC) represents a malignant neoplasm affecting the thyroid. Current treatment strategies include the removal of part of the thyroid; however, this approach is associated with a significant risk of developing hypothyroidism. In order to adequately understand the expression profiles of TNRC6C‐AS1 and STK4 and their potential functions in TC, an investigation into their involvement with Hippo signalling pathway and the mechanism by which they influence TC apoptosis and autophagy were conducted.

**Methods:**

A microarray analysis was performed to screen differentially expressed lncRNAs associated with TC. TC cells were employed to evaluate the role of TNRC6C‐AS1 by over‐expression or silencing means. The interaction of TNRC6C‐AS1 with methylation of STK4 promoter was evaluated to elucidate its ability to elicit autophagy, proliferation and apoptosis.

**Results:**

TNRC6C‐AS1 was up‐regulated while STK4 was down‐regulated, where methylation level was elevated. STK4 was verified as a target gene of TNRC6C‐AS1, which was enriched by methyltransferase. Methyltransferase’s binding to STK4 increased expression of its promoter. Over‐expressed TNRC6C‐AS1 inhibited STK4 by promoting STK4 methylation and reducing the total protein levels of MST1 and LATS1/2. The phosphorylation of YAP1 phosphorylation was decreased, which resulted in the promotion of SW579 cell proliferation and tumorigenicity.

**Conclusion:**

Based on our observations, we subsequently confirmed the anti‐proliferative, pro‐apoptotic and pro‐autophagy capabilities of TNRC6C‐AS1 through STK4 methylation *via* the Hippo signalling pathway in TC.

## 1 INTRODUCTION

As the most common malignancy of the endocrine organs, thyroid carcinoma (TC) often manifests itself as a tumour afflicting the thyroid epithelium. Studies have highlighted a rapid increase in TC incidence worldwide (>5% annually) with reports indicating that women tend to be much more susceptible than their male counterparts.^1^, ^2^ There are two differentiated subtypes of TC malignancies namely, follicular thyroid carcinoma and papillary thyroid carcinoma, both of which generally require extended periods to heal or to progress.^3^ Generally speaking, patients suffer from TC often undergo surgical treatment, including total thyroidectomy, and radioactive iodine both of which are widely considered to be a standard treatment option in clinical practice. That being said, both total thyroidectomy as well as radioactive iodine procedures can result in regional recurrence, which may lead to a reduction in life quality.^4^ Thus, new progressive therapies are required in order to more effectively target thyroid tumours. LncRNAs represent RNAs longer than 200 nucleotides, which have been linked to various biological processes, including that of nuclear import and RNA transcription. Our ever‐expanding knowledge of long non‐coding RNAs (lncRNAs) represents an opportunity to identify new therapeutic targets.

The altered expression of lncRNAs has been shown to be a critical process in thyroid carcinogenesis and has been linked to with patients having more adverse prognoses.^5^ The functions of lncRNAs have been documented in relation to their involvement in gene regulation at a post‐transcriptional level.^6^ For example, the lncRNA NAMA has been revealed as a downstream target gene of the mitogen‐activated protein kinase pathway with reports linking it to cellular‐growth arrest in papillary thyroid carcinoma.^7^ Serine/threonine‐protein kinase 4 (STK4), also referred to as mammalian sterile 20‐like kinase‐1 and Krs‐2, is a 56‐60‐kDa protein that has been shown to be down‐regulated in various cancers, including that of prostate cancer and hepatocellular carcinoma.^8^, ^9^ In addition, existing literature has suggested that DNA hypermethylation and post‐translational modification can mediate the loss of STK4 activity.^10^ Ready et al concluded that the dysregulation of STK4 is linked to carcinogenesis associated with poor outcomes in addition to implicating the Hippo signalling pathway in this process.^11^ The Hippo signalling pathway, comprised of mammalian STE20‐like protein kinase 1 and 2 (MST1/2), large tumour suppressor kinase 1/2 (LAST1/2) and its downstream transcription co‐activator, Yes‐associated protein (YAP), have been implicated in the regulation of tumour suppression concerning tissue repair and regeneration.^12^ Based on the exploration of the aforementioned literature, we asserted the hypothesis that lncRNA TNRC6C‐AS1 could potentially work in tandem with STK4 and the Hippo signalling pathway in TC cells, whereby tumour progression can be regulated.

## 2 MATERIALS AND METHODS

### 2.1 Ethics statement

The study was conducted with the approval of the Medical Ethics Committee of Suqian First Hospital and Huai’an Hospital Affiliated to Xuzhou Medical College and Huai’an Second People’s Hospital. All animal experiments were performed in strict accordance with the approval of the Animal Protection and Use Committee. All participants signed written informed consents.

### 2.2 Screening of differentially expressed genes in TC

Differentially expressed genes (DEGs) in TC were downloaded from the TCGA (http://cancergenome.nih.gov/) database, with R software employed for statistical analysis. Differential analysis was conducted for the transcriptome profiling data with package edgeR of R.^13^ False‐positive discovery (FDR) correction was performed on *P*‐values with the multitest package. FDR value of <0.05 and |log2 (fold change)| value of >2 were used as the threshold when screening out DEGs.

### 2.3 Study subjects

A total of 156 specimens (TC and adjacent normal tissues) were obtained from 78 patients with pathologically confirmed TC who had previously undergone surgical resection at the Suqian First Hospital and Huai’an Hospital Affiliated to Xuzhou Medical College and Huai’an Second People’s Hospital between October 2014 and October 2017. In accordance with the World Health Organization (WHO) classification of thyroid tumours,^14^ all cases in the current study were confirmed by means of morphological observation, while all diagnoses were made by two or more associate chief physicians from the Department of Pathology. Tumour staging was evaluated based on the *American Joint Committee on Cancer (AJCC) Cancer Staging Manual (6th ed.)*.^15^ Some of the specimens were fixed in 10% formaldehyde, conventionally dehydrated, embedded with paraffin and continuously sliced at a thickness of 4 μm for further use. The remaining samples were immediately placed in liquid nitrogen and stored at −80°C for further use.

### 2.4 Immunohistochemistry

The sliced tissue samples were incubated at 4°C overnight with a rabbit anti‐human STK4 primary antibody (ab97399, 1:300; Abcam, Cambridge, MA, USA). The slices were then washed three times with 0.1 mol/L phosphate buffer saline (PBS; 5 min/time). Following the addition of the goat anti‐rabbit IgG secondary antibody (ab6785, 1:1000; Abcam), incubation at 37°C for 20 minute was conducted, after which the slices were rinsed three times with PBS (5 min/time). A working solution of horseradish peroxidase (HRP)‐labelled streptavidin (0343‐10000U; Imunbio Co., Ltd., Beijing, China) was then added and incubated for 20 minute. The samples were then washed three times with 0.1 mol/L PBS, with diaminobenzidine (DAB, ST033; Whiga Co., Ltd, Guangzhou, Guangdong, China) subsequently added for coloration purposes. The colour reaction was observed under a microscope, and photographs of the slides were then acquired. Five high‐power visual fields were randomly selected with 100 cells in each field. The slices with positive cells <10% were considered to be negative, while the slices with 10% ≤positive cells <50% were considered to be positive. Furthermore, the slices with >50% positive cells were considered to be strongly positive.^16^

### 2.5 Methylation‐specific polymerase chain reaction

A Methyl Detector TM Bisulfite Modification kit (Active Motif Carlsbad, CA, USA) was applied for DNA methylation, followed by PCR amplification. The primers for STK4 MSP‐M and STK4 MSP‐U were synthesized by Sangon Biotech (Shanghai, China), as illustrated in Table 1. The reaction conditions employed were as follows: pre‐denaturation at 95°C for 10 minute, and 35 cycles of denaturation at 94°C for 1 minute, annealing at 60°C for 50 second and extension at 72°C for 10 minute. PCR products were resolved using a 20 g/L agarose. The results obtained were then observed using an automatic analysis system of electrophoresis gel imaging. Due to the fact that the methylated primers could amplify products while the unmethylated primers could not amplify products, the primers were identified as fully methylated, fully unmethylated and partly unmethylated.^17^ The aforementioned method was applicable in an identical fashion to the detection among the cells. Each experiment was repeated three times.

**Table 1:** Primer sequences for RT‐qPCR

Gene	Sequence (5′‐3′)
TNRC6C‐AS1	F: GAAATGGTCAAAGAGCGATGGG R: CACGTCACTTTCTGGACTGC
STK4	F: GGTCAAGATTGCTGAGTGAGTG R: TCACAGATGGAGAGCCGAGT
Bax	F: CGGGAGATGCTCATTGGACA R: TGACTCAGATGCCGAAGTGTG
Bcl‐2	F: CTTTGAGTTCGGTGGGGTCA R: AGCCCAGACTCACATCACCA
Beclin‐1	F: TCCGGGCTCCCGAGG R: GCTGTTGGCACTTTCTGTGG
LC3	F: AGTAACCCACGCGATTGTGAT R: TGCGGAAGAATGGAACACCC
STK4 MSP‐M	F: GTTCGAATTACGTGATTAGGGTC R: CTCAACTATACCGTCTCCATAACG
STK4 MSP‐U	F: GTTTGAATTATGTGATTAGGGTTGT R: CCTCAACTATACCATCTCCATAACAC
GAPDH	F: GCACCGTCAAGGCTGAGAAC R: TGGTGAAGACGCCAGTGGA

Bax, Bcl‐2‐associated protein X; Bcl‐2, B‐cell lymphoma‐2; GAPDH, glyceraldehyde‐3‐phosphate dehydrogenase; LC3, light chain 3; RT‐qPCR, Reverse transcription quantitative polymerase chain reaction; STK4, serine/threonine‐protein kinase 4.

### 2.6 Reverse transcription quantitative PCR

Total RNA was extracted from both the TC and adjacent normal tissues using a TRIZOL kit (15596‐018; Beijing Solarbio Science & Technology Co., Ltd., Beijing, China) in strict accordance with the manufacturer's instructions. RNA concentration was determined using a spectrometer. The primers were synthesized by Takara Biotechnology Co., Ltd. (Dalian, Liaoning, China; Table 1). Reverse transcription was performed based on the provided manufacturer's instructions for the cDNA RT kit (K1622; Beijing Yaanda Biotechnology Co., Ltd., Beijing, China). The reaction conditions were as follows: at 42°C for 30‐50 minute (reverse transcription reaction) followed by 85°C for 5 second (reverse transcriptase inactivation reaction). The obtained cDNA was diluted to 50 ng/μL for subsequent quantitative PCR purposes. The amplification system of qPCR was set as 25 μL. RT‐qPCR was performed in a qPCR instrument (ViiA 7; Daan Gene Co., Ltd., Guangzhou, Guangdong, China). The RT reaction conditions were as follows: pre‐denaturation at 95°C for 4 minute, and 30 cycles of denaturation at 95°C for 30 second, annealing at 57°C for 30 second and extension at 72°C for 30 second. A total of 2 μg cDNA was utilized as a qPCR template while β‐actin was regarded as the internal reference. The 2^−ΔΔCt^ method was applied in order to calculate the relative expression of the target genes (TNRC6C‐AS1 and STK4), apoptosis‐related factors (Bcl‐2 associated protein X [Bax] and B‐cell lymphoma‐2 [Bcl‐2]) and autophagy‐related factors (Beclin‐1 and light chain 3 [LC3]). The formula employed was as follows: ΔCt = Ct_target gene_ − Ct_internal reference_ 18 This method was applicable in an equal fashion to the RT‐qPCR detection of the cells. Each experiment was repeated three times.

### 2.7 Western blot analysis

After the total protein had been extracted from both TC and adjacent normal tissues, the protein concentration of each sample was determined using a bicinchoninic acid (BCA) kit (20201ES76; Yeasen Biotechnology Co., Ltd., Shanghai, China). The extracted proteins were run on polyacrylamide gel electrophoresis (PAGE) for separation purposes. The proteins on the gel were subsequently transferred by wet method onto a polyvinylidene fluoride (PVDF) membrane. The membrane was then blocked with 5% bovine serum albumin (BSA) at room temperature for 1 hour and then incubated with the following primary antibodies on a shaker at 4°C overnight: STK4 (ab97399, 1:2000), MST1 (ab96705, 1:2000), LATS1 (ab70565, 1:2000), LATS2 (ab70565, 1:2000), YAP1 (ab62751, 1:1000), p‐YAP1 (ab62751, 1:1000), Bax (ab53154, 1:800), Bcl‐2 (ab59348, 1:800), Beclin‐1 (ab217179, 1:1000) and LC3 (ab51520, 1:3000). After Tris‐Buffered Saline Tween‐20 (TBST) had been used to wash the membrane for three times (5 min/time), HRP‐labelled goat anti‐rabbit IgG (ab205718, 1:20000) was added for further incubation at room temperature for 1 hour. Afterwards, the membrane was washed three times (3 min/time) with TBST. Next, a developer was utilized for coloration purposes. All employed antibodies were purchased from Abcam Inc. Quantitative analysis was conducted using ImageJ 1.48u software (National Institutes of Health, Bethesda, MD, USA). Glyceraldehyde‐3‐phosphate dehydrogenase (GAPDH) was considered as the internal protein reference, while the ratio of the grey value of the target band to the internal reference band was used to calculate the relative expression of the target protein. This method was equally applicable to the Western blot detection of the cells. Each experiment was repeated three times.

### 2.8 Cell culture

Human normal thyroid cell line Nthy‐ori 3‐1 and TC cell lines FRO, ARO, PDTC‐1 and SW579 (Cell Bank of China Center for Type Culture Collection, Shanghai, China) were cultured in a 1640 medium containing 10% foetal bovine serum and penicillin‐streptomycin solution (1:1; final concentration: 100 U/mL). Following incubation at 37°C with 5% CO_2_, RT‐qPCR was performed in an attempt to select the cell line with the highest expression levels of lncRNA TNRC6C‐AS1. Cells at the logarithmic growth stage were selected for subsequent experimentation.

### 2.9 Cell grouping and transfection

Thyroid carcinoma SW579 cells displaying logarithmic growth were seeded into a 6‐well plate. When cell confluence reached 80%‐90%, lncRNA RP11‐468E2.5 shRNA (Santa Cruz Biotechnology, Dallas, TX, USA) sequences and X‐tremeGENE shRNA transfection agent were diluted using 50 μL of 1640 medium and fully mixed together. After incubation for 20 minute, the mixture was added into a 6‐well plate. Following transfection, the plate was incubated under saturated humidity atmosphere conditions at 37°C with 5% CO_2_ over a period of 48 hour. The medium containing the transfection solution was discarded, and cells were further cultured for 24‐48 hour in an RPMI 1640 medium (Santa Cruz Biotechnology) containing 10% foetal bovine serum. The cultured cells were assigned into the following groups: OE‐TNRC6C‐negative control (NC; cells transfected with OE‐TNRC6C NC plasmids), OE‐TNRC6C (cells transfected with OE‐TNRC6C plasmids), si‐TNRC6C‐NC (cells transfected with si‐TNRC6C NC plasmids), si‐TNRC6C (cells transfected with si‐TNRC6C plasmids), OE‐TNRC6C + dimethyl sulfoxide (DMSO; cells co‐transfected with OE‐TNRC6C plasmids + 5‐Aza‐CdR solution, DMSO), OE‐TNRC6C + 5‐Aza‐CdR (cells co‐transfected with OE‐TNRC6C plasmids + DNA methylation inhibitors, 5‐Aza‐CdR), NC (cells transfected with OE‐STK4 NC plasmids), OE‐STK4 (cells transfected with OE‐STK4 plasmids), si‐NC (cells transfected with si‐STK4 NC plasmids), si‐STK4 (cells transfected with si‐STK4 plasmids), OE‐STK4 + DMSO (cells co‐transfected with OE‐STK4 plasmids + Verteporfin solution DMSO) and OE‐STK4 + Verteporfin (cells co‐transfected with OE‐STK4 plasmids + the Hippo signalling pathway inhibitors Verteporfin). All the above sequences and plasmids were synthesized by Sangon Biotech. After 48 hour of transfection, transfected cells were observed under a fluorescence microscope in order to confirm successful transfection.^19^ A transfection efficiency of >75% was considered to be reflective of successful transfection.

### 2.10 Fluorescence in situ hybridization

The localization of TNRC6C‐AS1 expression in SW579 cells was predicted using the website http://lncatlas.crg.eu/. TNRC6C‐AS1 sub‐cellular localization was detected using a fluorescence in situ hybridization (FISH) kit (Roche, Basel, Switzerland). Fluorescence images were obtained under a confocal laser scanning microscope (FV1000; Olympus, Tokyo, Japan). Each experiment was repeated three times.

### 2.11 Luciferase reporter gene assay

The existence of complementary base pairing binding sites between TNRC6‐AS1 and STK4 promoter region was identified in accordance with the online bioinformatics analysis software BLAST (https://blast.ncbi.nlm.nih.gov/Blast.cgi?PAGE=MegaBlast&PROGRAM=blastn&BLAST_PROGRAMS=megaBlast&PAGE_TYPE=BlastSearch&BLAST_SPEC=blast2seq&DATABASE=n/a). The above prediction of TNRC6C‐AS1 was further validated by means of a luciferase reporter gene assay. Dual‐luciferase reporter gene vectors, pGLO‐STK4 wide type (wt) and pGLO‐STK4 mutant type (mut), were constructed. The luciferase reporter plasmids were co‐transfected into HEK cells with an over‐expressed pcDNA‐TNRC6C‐AS1 plasmid or a NC plasmid. The cells were then lysed at 48 hour after transfection, followed by centrifugation at 25,764 × *g* for 1 minute and subsequent supernatant collection. The luciferase activity in the transfected cells was measured using a Dual‐Luciferase^®^ Reporter Assay System (E1910; Promega Corp., Madison, WI, USA). Relative luciferase activity = firefly luciferase activity/renilla luciferase activity. Each experiment was conducted three times.

### 2.12 RNA‐binding protein immunoprecipitation

The experiment was performed in accordance with the instructions of the Magna RNA‐binding protein immunoprecipitation (RIP) RNA‐Binding Protein Immunoprecipitation kit (Millipore, Billerica, MA, USA). Specific antibodies for the target proteins included rabbit anti‐DNMT1 (ab13537), rabbit anti‐DNMT3a (ab2850) and rabbit anti‐DNMT3b (ab2851). All the specific antibodies were purchased from Abcam Inc. The relevant RNA was extracted and purified by means of precipitation. The interaction of DNA methyltransferase1 (DNMT1), DNMT3a and DNMT3b with LINC00261 was verified by RT‐qPCR using TNRC6C‐AS1‐specific primers as depicted in Table 1.

### 2.13 Chromatin immunoprecipitation

The enrichment analysis of DNMT1, DNMT2 and DNMT3 in STK4 promoter region was conducted using a chromatin immunoprecipitation (CHIP) kit (Millipore). The binding of DNMT1, DNMT2 and DNMT3 to STK4 promoter region was detected using specific primers in STK4 promoter region (Table 1). Each experiment was repeated three times.

### 2.14 Immunofluorescence staining

Following conventional detachment and transfection, the cells were counted and cultured in immunofluorescence chambers at a density of 2 × 10^5^ cells/well. When confluence had reached approximately 90%, the cells were washed three times with PBS on ice and fixed in 4% paraformaldehyde (1 mL/well) at room temperature for 15 minute, followed by three additional PBS washes. The cells were then treated with 0.3% Triton for 10 minute and washed three times with PBS. Cell blockade was conducted in goat serum for 30 minute and incubated with the following primary antibodies prepared using PBS at 4°C overnight: rabbit anti‐human MST1 (ab96705, 1:150), LATS1 (ab191022, 1:200) and YAP1 (ab62751, 1:200). After three PBS washes, goat anti‐rabbit IgG (ab6785, 1:1000) was added as the secondary antibody for incubation at room temperature for 1 hour under conditions void of light. All antibodies were purchased from Abcam Inc. The unbound antibody was removed by means of three PBS washes. In the next step, 4′, 6‐diamidino‐2‐phenylindole (DAPI) was added to stain the cells over a period of 15 minute. The cells were then washed three times with PBS under dark conditions and sealed with a fluorescent quencher. Photographs were subsequently obtained under the guidance of a fluorescence microscope. Each experiment was repeated three times.

### 2.15 3‐(4,5)‐dimethylthiahiazo (‐z‐y1)‐3,5‐di‐phenytetrazoliumromide assay

When cell density had reached approximately 80% following transfection, the SW579 cells were washed twice with PBS, detached with 0.25% trypsin, and a single cell suspension was then prepared. After tallying, the cells were seeded into a 96‐well plate at a cell density of 3 × 10^3^ to 6 × 10^3^ cells/well (200 μL; with 6 repeating wells). After 48 hour, 20 μL of a 5 mg/mL 3‐(4,5)‐dimethylthiahiazo (‐z‐y1)‐3,5‐di‐phenytetrazoliumromide (MTT) solution (A2776‐1g, Shanghai Shengfeng Biological Technology Co., Ltd., Shanghai, China) was added into each well. After an additional 4 hour of incubation, the culture solution was aspirated and 150 μL DMSO was added to each well and gently shaken for 10 minute. The optical density (OD) values at 490 nm were measured using an enzyme‐linked immunometric metre at the 12, 24 and 48 hour time points, respectively. With time points as the abscissa and the OD value as the ordinate, a cell viability curve was plotted. Each experiment was conducted three times.

### 2.16 5‐Ethynyl‐2’‐deoxyuridine assay

Thyroid carcinoma cells at the logarithmic growth phase were seeded into a 96‐well plate with 2 × 10^3^ to 4 × 10^4^ cells in each well, which were then permitted to attach over a 24 hour period. Transfection was performed with triplicate wells in each group. Next, 100 μL fixative was added into each well and incubated for 30 minute at room temperature. Next, 2 mg/mL glycine was added into each well for another 5 minute of incubation. The plate was then washed with PBS (100 μL/well) for 5 minute. A penetrant (100 μL/well, PBS containing 0.5% Triton X‐100) was subsequently added and incubated for 10 minute, followed by a series of PBS washes. Next, the cells were incubated following the addition of 1× Apollo staining solution for 30 minute under conditions void of light. After penetrant addition and methanol washing, 100 μL of 1× Hoechst 33342 reaction solution was added to each well and incubated at room temperature for 30 minute under dark conditions for decolorization purposes. Afterwards, the cells were washed three times with PBS (100 μL/well). Finally, the cells were sealed with a fluorescent quenching mounting medium (100 μL/well) and observed under a fluorescence microscope.

### 2.17 Flow cytometry

The apoptosis of the SW579 cells following a 24 hour period of treatment was detected using an Annexin V‐fluorescein isothiocyanate (FITC)/propidium iodide (PI) double staining kit (556547, Shanghai Surejia Biotech Co., Ltd., Shanghai, China). After mixing with Annexin V‐FITC, the cells were incubated at room temperature for 15 minute under conditions void of light. Next, 5 μL of PI was added to each sample and ice bathed for 5 minute under dark conditions. Finally, FITC was detected at an excitation wavelength of 488 nm as well as an emission wavelength of 530 nm, with PI detected at >575 nm using flow cytometry (Cube6, Partec, Germany). Each experiment was repeated three times.

### 2.18 Hoechst staining

A coverslip was placed on the 6‐well plate. Following a 24 hour of treatment, HCT116 and SW579 cells were seeded into the 6‐well plate at the density of 5 × 10^5^ cells/well and cultured in an incubator overnight at 37°C with 5% CO_2_. When cell confluence had reached approximately 50%‐80%, the cells were fixed with 0.5 mL fixative for 10 minute on the following day. After the fixative had been removed, the cells were washed twice with PBS. After the removal of liquid, the samples were stained with 0.5 mL Hoechst 33258 dye (0.5 μg/mL) for 5 minute and then washed twice with PBS. Finally, a drop of fluorescent quenching mounting medium was added dropwise on the glass slide, which was then covered with a cell‐possessed coverslip. Cellular morphology was analysed, and the blue‐stained nuclei were observed under a fluorescence microscope. Each experiment was repeated 3 times.

### 2.19 Monodansylcadaverine staining

An autophagy inhibitor, 3‐MA, was diluted using sterile PBS culminating in a final concentration of 40 mmol/L, aliquoted and stored at 20ºC. Monodansylcadaverine (MDC) powder (Sigma‐Aldrich Chemical Company) was dissolved using DMSO in order to reach a final concentration of 0.1 mol/L and stored at −20ºC. The SW579 cells in each group were seeded into a 6‐well plate, treated with 3‐MA and cultured in a medium containing 50 μm MDC at 37°C with 5% CO_2_ for 10 minute, followed by centrifugation at 178 × *g* (10 minute each time). After re‐suspension in PBS, the cells were placed on a glass slide and covered with a coverslip. An inverted fluorescence microscope OLYMPUS IX71 (Olympus Optical) was then employed to observe the cells under excitation using ultraviolet light.

### 2.20 Observation under a transmission electron microscope

After the cells had been collected, they were then fixed in a 2.5% glutaraldehyde solution at 4°C overnight and washed four times with 0.1 mol/L PBS. After aspirating the PBS, the cells were fixed with osmic acid at 4°C for 1 hour, dehydrated with gradient acetone (50%, 70%, 90% and 100%) and subsequently dried. The embedded cells were then polymerized in an oven prior to the preparation of electron microscopic serial sections. During transmission electron microscope (TEM) analysis, the slices were placed on a single‐sample rod in a JEMI400 TEM and photography at an accelerating voltage of 80 kV. The gold standard of autophagy was considered to be the existence of autophagosomes within the cytoplasm. Under a TEM, a classic autophagosome globe is wrapped by double or multiple membranes comprised of materials to be degraded. Each experiment was done three times.

### 2.21 Xenograft tumour in nude mice

A total of 48 BALB/c nude mice (age: 4‐6 weeks, weight: 16‐20 g, Shanghai Lingchang Biotech Co., Ltd.) were evenly assigned into the following groups: OE‐TNRC6C‐NC, OE‐TNRC6C, si‐TNRC6C‐NC, si‐TNRC6C, OE‐TNRC6C + NC and OE‐TNRC6C + OE‐STK4. The transfected cells displaying logarithmic growth were made into cell suspension at a concentration of 5 × 10^7^ cells/mL. The subcutaneous part of the left axilla of each mouse was injected with 0.2 mL of cell suspension using a 1 mL syringe. All mice were fed in a specific pathogen‐free (SPF) animal facility under a laminar flow hood and sacrificed on the 7, 14, 21 and 28 days, respectively. Tumour growth was observed and recorded at regular intervals. The maximum diameter (a) and minimum diameter (b) of the tumours in mice were measured using a ruler. The tumour volume was calculated according to *V* (mm^3^) = π (*a* × *b* ^2^)/6. All measurements were repeated three times.

### 2.22 Statistical analysis

All data were processed by SPSS 21.0 statistical software (IBM Corp. Armonk, NY, USA). Measurement data were expressed as mean ± standard deviation. The *t* test was used to compare data between two groups. One‐way analysis of variance (ANOVA) was employed for comparisons among multiple groups. A *P* value of <0.05 was considered to be indicative of statistical significance.

## 3 RESULTS

### 3.1 LncRNA TNRC6C‐AS1 was highly expressed while STK4 was reduced in TC tissues

**Figure 1:**
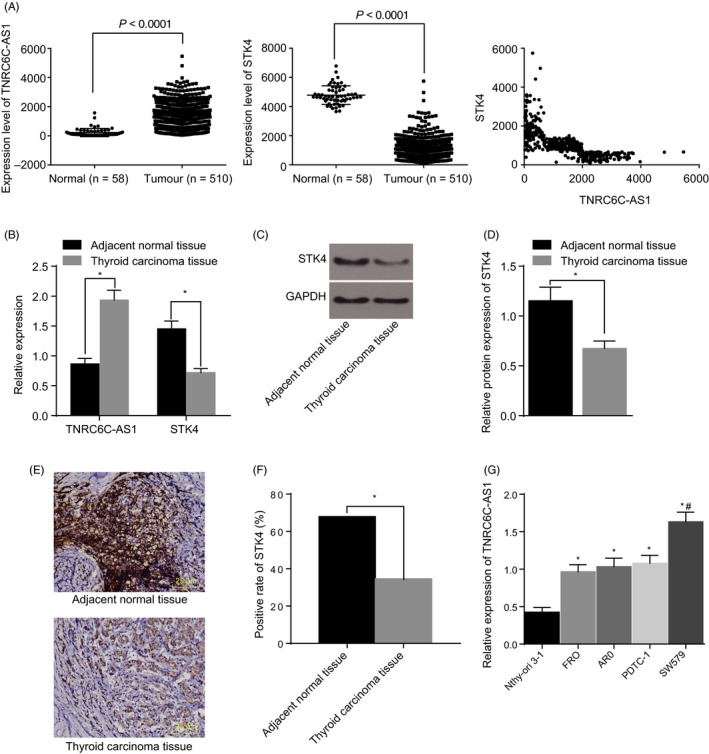
LncRNA TNRC6C‐AS1 was highly expressed while STK4 is poorly expressed in thyroid carcinoma (TC) tissues. Panel A, TCGA database screening and correlation analysis of TNRC6C‐AS1 and STK4 expression in TC. Panel B, relative expression of TNRC6C‐AS1 and STK4 in adjacent normal and TC tissues as determined by RT‐qPCR; the data expressed as mean ± standard deviation and analysed using *t* test; N = 78. Panel C, D, relative protein expression of TNRC6C‐AS1 and STK4 in adjacent normal and TC tissues as determined by Western blot analysis; the data were expressed as mean ± standard deviation and analysed using *t* test; N = 78. Panel E, F, positive rate of STK4 in adjacent normal and TC tissues as detected by immunohistochemistry (200×); the data were analysed using chi‐square test; N = 78. Panel G, relative expression of TNRC6C‐AS1 in various TC cell lines, and the cell line with the highest TNRC6C‐AS1 expression was selected for subsequent experiments; the data were expressed as mean ± standard deviation and analysed using one‐way ANOVA; N = 78. *P < 0.05 vs. adjacent normal tissue or Nthy‐ori 3‐1 cell line. ^#^The highest TNRC6C‐AS1 expression

Initially, based on the analysis results of The Cancer Genome Atlas (TCGA), TNRC6C‐AS1 was determined to be highly expressed in TC tissues while STK4 was poorly expressed. As depicted in Figure 1A, A negative correlation was found between TNRC6C‐AS1 and STK4. RT‐qPCR and Western blot analysis methods were performed in order to determine the expression levels of lncRNA TNRC6C‐AS1 and STK4 in TC and adjacent normal tissues. The results revealed notably elevated lncRNA TNRC6C‐AS1 expressions while the levels of STK4 were decreased in TC tissues when compared the adjacent normal tissues (*P < *0.05; Figure 1B‐D). Next, an immunohistochemistry assay was performed in order to detect the STK4‐positive expression, the results of which indicated that the positive expression of STK4 was primarily in the cytoplasm, while the positive rate of STK4 was much lower in the TC tissues when compared to the adjacent normal tissues (*P < *0.05; Figure 1E,F). RT‐qPCR methods were further conducted in an attempt to select the TC cell line with the highest TNRC6C‐AS1 expression. Compared with normal thyroid cell line Nthy‐ori 3‐1, the relative expression of TNRC6C‐AS1 elevated in TC cell lines FRO, ARO, PDTC‐1 and SW579, among which the cell line SW579 exhibited the highest TNRC6C‐AS1 expression (*P < *0.05; Figure 1G). On the basis of the aforementioned findings, the TC cell line SW579 was selected for subsequent experiments.

### 3.2 STK4 was a downstream target gene of TNRC6C‐AS1

**Figure 2:**
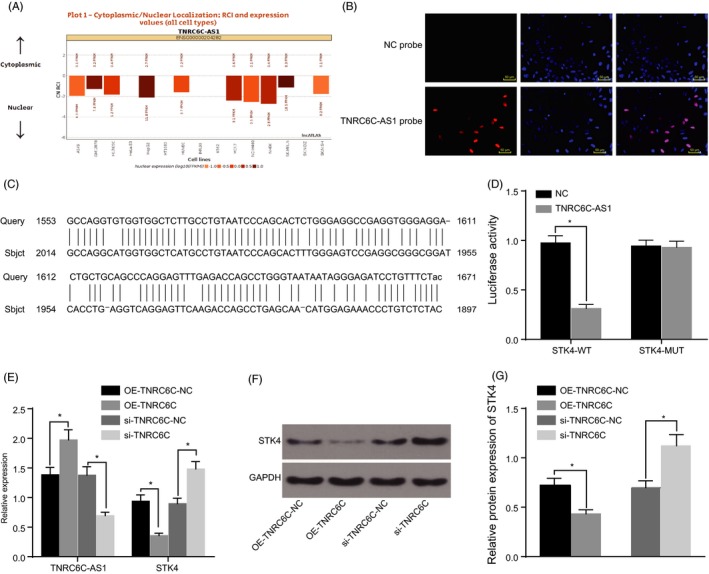
STK4 was a downstream target gene of TNRC6C‐AS1. Panel A, localization of TNRC6C‐AS1 in cells as predicted on http://lncatlas.crg.eu/, indicating that TNRC6C‐AS1 was located in the nucleus. Panel B, TNRC6C‐AS1 localization in the nucleus, as verified by FISH (200×). Panel C, TNRC6C‐AS1 binds to the STK4‐3'UTR, as predicted by the online target prediction software. Panel D, the luciferase activity of cells was decreased after the treatment by a combination of Wt‐TNRC6C‐AS1 and STK4, suggesting that STK4 was a target gene of TNRC6C‐AS1. Panel E, RT‐qPCR was used to determine the relative expression of TNRC6C‐AS1 and STK4 after the transfection. Panel F, G, Western blot analysis was used to determine the relative protein expression of STK4 after the transfection. *P < 0.05 vs*. *the OE‐TNRC6C‐NC and si‐TNRC6C‐NC groups; the data are expressed as mean ± standard deviation and analysed using *t* test and one‐way ANOVA; the experiment was repeated three times

The sub‐cellular localization of TNRC6C‐AS1 was predicted using the site http://lncatlas.crg.eu/, which revealed that TNRC6C‐AS1 was located in the nucleus of multiple cell lines (Figure 2A). This finding was further verified by experimental FISH means, which demonstrated that TNRC6C‐AS1 was primarily expressed in the nucleus (Figure 2B).

Furthermore, we used an online bioinformatics analysis software Blast and luciferase assays to examine whether TNRC6C‐AS1 could directly regulate STK4 expression. The results of online bioinformatics analysis software revealed the existence of complementary base pairing binding sites between TNRC6C‐AS1 and STK4 promoter region (Figure 2C), suggesting that STK4 was indeed a target gene of TNRC6C‐AS1. The targeting relationship between STK4 and TNRC6C‐AS1 was further verified through the application of the luciferase reporter gene assay (Figure 2D). The results indicated that the luciferase activity of STK4 wild type was significantly inhibited in the TNRC6C‐AS1 group when compared to that in the NC group (*P* < 0.05), while the luciferase activity of mutant 3′‐UTR did not exhibit any significant change (*P* > 0.05). These results indicated that TNRC6C‐AS1 could directly target STK4.

In order to ascertain as to whether TNRC6C‐AS1 could influence STK4 expression, RT‐qPCR and Western blot analysis methods were conducted following transfection, the results of which are shown in Figure 2E‐G. No significant difference was observed in relation to the expression of STK4 detected after cell transfection between the OE‐TNRC6C‐NC and si‐TNRC6C‐NC groups (*P* > 0.05). Compared with the OE‐TNRC6C‐NC group, TNRC6C‐AS1 was up‐regulated but STK4 was markedly down‐regulated in the OE‐TNRC6C group (*P* < 0.05). Compared with the si‐TNRC6C‐NC group, the opposite tendency was observed in the si‐TNRC6C group (*P* < 0.05). All the obtained data indicated that STK4 expression could be regulated by TNRC6C‐AS1.

### 3.3 TNRC6C‐AS1 inhibited STK4 expression through DNA methylation

**Figure 3:**
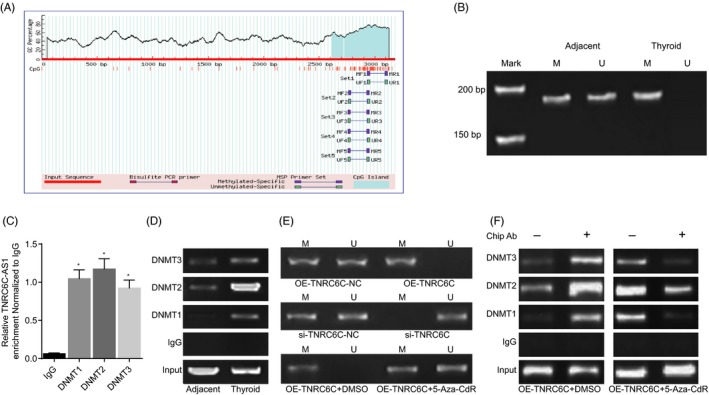
TNRC6C‐AS1 inhibited STK4 expression through DNA methylation. Panel A, the promoter region of STK4 was located in the CpG island, as shown by MethPrimer. Panel B, electrophoretogram illustrating the methylation level of STK4 in thyroid carcinoma (TC) tissues as detected by MSP. Panel C, TNRC6C‐AS1 enrichment by methyltransferase as verified by RIP. Panel D, the relative methyltransferase enrichment in STK4 promoter region in adjacent normal and TC tissues as detected by ChIP. Panel E, electrophoretogram illustrating the methylation level of STK4 in each group as detected by MSP. Panel F, the relative methyltransferase enrichment in STK4 promoter region after demethylation of STK4 by 5‐Aza‐CdR treatment, as detected by CHIP. *P < 0.05 vs*. *the IgG group; the data are expressed as mean ± standard deviation and analysed using one‐way ANOVA; the experiment was repeated three times

MethPrimer software was applied for the analysis of CpG island in STK4 promoter region by inputting nucleotide sequence around 3000 bp close to the STK4 promoter region. The results revealed that STK4 promoter region was located in the CpG island (Figure 3A), indicating that STK4 expression was indeed affected by promoter methylation. In order to verify as to whether TNRC6C‐AS1 was associated with the methylation level of STK4 promoter region, 78 cases of TC samples were randomly selected with the methylation level of the CpG island in the STK4 promoter region of the selected samples were detected using methylation‐specific polymerase (MSP). The results revealed that the methylation level of STK4 was notably elevated in the TC tissues compared to that of the adjacent normal tissues (*P < *0.05; Figure 3B). The obtained results suggested that the STK4 expression decreased as the methylation level increased, which provided evidence proving that STK4 was poorly expressed in TC tissues (Table 2). RIP was then employed to detect the relative TNRC6C‐AS1 enrichment binding to IgG, DNMT1, DNMT2 and DNMT3, respectively. After being normalized to IgG, the relative TNRC6C‐AS1 enrichment binding to DNMT1, DNMT2 and DNMT3 was notably increased, which demonstrated that TNRC6C‐AS1 promoted DNMT1, DNMT2 and DNMT3 enrichment (Figure 3C). CHIP was then performed in order to detect the relative methyltransferase enrichment in STK4 promoter region in adjacent normal and TC tissues. The results revealed the existence of methyltransferase enrichment in the STK4 promoter region in TC tissues, while limited enrichment was observed in the adjacent normal tissues (*P < *0.05) (Figure 3D).

**Table 2:** Methylation level of STK4 in thyroid carcinoma and adjacent normal tissues

Group	N	STK4			
		Unmethylated	Unmethylation level (%)	Methylated	Methylation level (%)
Thyroid carcinoma tissue	78	14	17.95*	64	82.05*
Adjacent normal tissue	78	59	75.64	19	24.36

STK4, serine/threonine‐protein kinase 4.

* *P* < 0.05 vs. adjacent normal tissue; measurement data are analysed by *t* test; N = 78; the experiment was repeated three times.

MSP was employed in order to detect the methylation level of CpG island on STK4 promoter in TC cells with either highly expressed or poorly expressed TNRC6C‐AS1. The results revealed that the CpG island in STK4 possessed a higher methylation level in the cells with over‐expressed TNRC6C‐AS1 while reductions in STK4 expression were noted, vice versa. DNA methyltransferase inhibitor 5‐Aza‐CdR was introduced to promote the demethylation of STK4 promoter region in order to investigate the regulatory mechanism between TNRC6C‐AS1 and SKT4. The obtained MSP results revealed that STK4 methylation was higher in the OE‐TNRC6C + DMSO group but lower in the OE‐TNRC6C + 5‐Aza‐CdR group (*P < *0.05; Figure 3E). Through CHIP, an evident reduction was found in relation to the relative methyltransferase enrichment in STK4 promoter region of the cells in the OE‐TNRC6C + 5‐Aza‐CdR group compared with the OE‐TNRC6C + DMSO group, inferring that 5‐Aza‐CdR could reverse the STK4 methylation caused by TNRC6C‐AS1 over‐expression in TC cells (Figure 3F). The above‐mentioned findings provided elucidated indicating that the methylation of CpG island in the STK4 promoter region shares a significant correlation to TNRC6C‐AS1 expression.

### 3.4 STK4 modulated the Hippo signalling pathway

**Figure 4:**
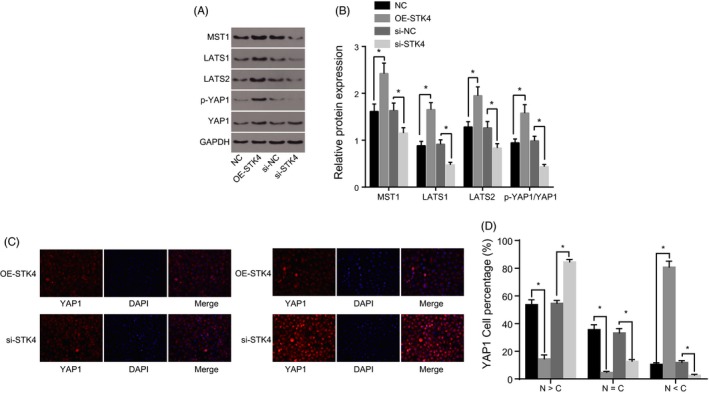
STK4 modulated the Hippo signalling pathway. Panel A, B, Western blot analysis is used to determine the relative protein expression of factors related to the Hippo signalling pathway (MST1, LAST1, LAST2, YAP1 and p‐YAP1) in each group. Panel C, localization of YAP1 in each group after the transfection as detected by immunofluorescence staining (400×). Panel D, percentage of YAP1 in the nucleus/cytoplasm as detected by immunofluorescence staining. *P < 0.05 vs*. *the NC and si‐NC groups; the data are expressed as mean ± standard deviation and analysed using one‐way ANOVA; the experiment was repeated three times

The effects of STK4 on the Hippo signalling pathway were investigated through the application of a Western blot analysis (Figure 4A,B). The results revealed that compared with the NC group, the OE‐STK4 group exhibited elevated levels of MST1, LAST1 and LAST2, as well as increased YAP1 phosphorylation (*P < *0.05). An opposite trend was observed in the si‐STK4 group (*P < *0.05).

Immunofluorescence staining was performed in order to detect the nuclear transfer of YAP1 in SW579 cells. As illustrated in Figure 4C,D, YAP1 displayed nuclear transfer with red‐stained YAP1 and blue‐stained nucleus. The localization of YAP1 under the Merge operation was clear. Compared with the NC group, the YAP1 expression decreased remarkably in the OE‐STK4 group (*P < *0.05), with more YAP1 translocated from nucleus to the cytoplasm. In addition, in comparison with the si‐NC group (*P < *0.05), enhanced YAP1 activity was detected in the si‐STK4 group, which exhibited increased translocation of YAP1 from the cytoplasm to the nucleus. The aforementioned results demonstrated that silencing of STK4 could inhibit MST1 and LAST1 expression, and reduce YAP1 phosphorylation, ultimately contributing to the nuclear transfer of YAP1.

### 3.5 Up‐regulation of STK4 inhibited proliferation while promoting apoptosis and autophagy via the Hippo signalling pathway

**Figure 5:**
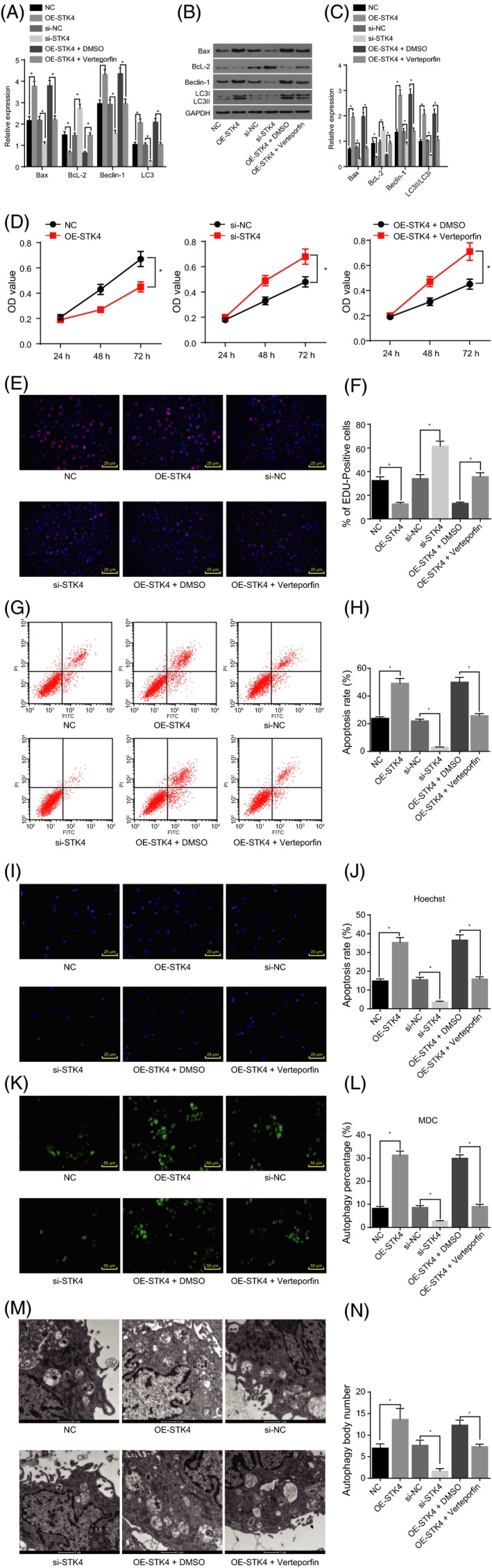
Up‐regulation of STK4 inhibited thyroid carcinoma (TC) cell proliferation while promoting apoptosis and autophagy *via* the Hippo signalling pathway. Panel A, RT‐qPCR was employed to determine the relative expression of apoptosis‐related factors (Bax and Bcl‐2) and autophagy‐related factors (Beclin‐1 and LC3). Panel B, C, Western blot analysis was used to determine the relative protein expression of apoptosis‐related factors (Bax and Bcl‐2) and autophagy‐related factors (Beclin‐1, LC3I, LC3II and LC3II/LC3I). Panel D, MTT assay was used to measure the OD values in each group. Panel E, F, EdU assay is used to stain apoptotic cells in each group (200×). Panel G, H, flow cytometry is used to detect the apoptosis of TC cells in each group. Panel I, J, Hoechst staining was used to detect the apoptosis of TC cells in each group (400×). Panel K, L, MDC staining is used to detect the autophagic vacuole of TC cells in each group (200×). Panel M, N, TEM was used to detect the autophagic vacuole of TC cells in each group (15 000×). *P < 0.05 vs*. *the NC, si‐NC and OE‐STK4 + DMSO groups; the data are analysed using one‐way ANOVA; the experiment was repeated three times

RT‐qPCR and Western blot analysis were conducted to determine the expression of factors related to TC cell proliferation, apoptosis and autophagy. As depicted in Figure 5A‐C in comparison with the NC group, the OE‐STK4 group displayed higher expression levels of Bax, Beclin‐1 and LC3, but a lower Bcl‐2 level (*P < *0.05). The si‐STK4 group exhibited a contrasting trend when compared with the si‐NC group (*P < *0.05). Cell proliferation was evaluated using MTT and 5‐Ethynyl‐2’‐deoxyuridine (EdU) assays, and the results are shown in Figure 5D‐F. At the same cell concentration, the OD values at 24 hour post‐transfection were not determined to be significantly different among the groups (*P* > 0.05). Compared with those in the NC group, the OD values at 48 and 72 hour as well as the number of red‐stained apoptotic cells decreased in the OE‐STK4 group (*P < *0.05). Compared with the si‐NC group, the si‐STK4 group exhibited much higher OD values at 48 and 72 hour, with a greater presence of red‐stained apoptotic cells (*P < *0.05). Flow cytometry and Hoechst staining were employed to detect cell apoptosis. As shown in Figure 5G‐J, the apoptotic rate in the OE‐STK4 group was higher than that in the NC group (*P < *0.05), while the OE‐STK4 group exhibited more distinct pyknotic, broken and white nuclei. A reduced apoptotic rate was found in the si‐STK4 group, which was accompanied by a reduced number of pyknotic, broken and white nuclei (*P < *0.05). Furthermore, cell autophagy was assessed using MDC fluorescent staining and TEM. The results (Figure 5K‐N) revealed that the cells in the NC and si‐NC groups exhibited sound morphology with evenly light green‐stained cytoplasm as well as less green fluorescence (autophagy indicator). Compared with the NC group, a greater degree of cell shrinkage as well as more green fluorescence in the OE‐STK4 group was detected. In addition, double‐layered autophagic vacuoles were observed under the TEM. In accordance with the TEM observations, the cells in the si‐STK4 group displayed an intact cellular morphology with less green fluorescence and fewer double‐layered autophagic vacuoles when compared to the si‐NC group.

The Hippo signalling pathway may be involved in inducing various cellular processes. In an attempt to evaluate this possibility, the cells were treated with inhibitors of the Hippo signalling pathway. Compared with the OE‐STK4 + DMSO group, the expression of Bax, Beclin‐1 and LC3 was diminished, while the Bcl‐2 level was elevated in the OE‐STK4 + Verteporfin group (Figure 5A‐C). Furthermore, increased proliferation (Figure 5D‐F), repressed apoptosis (Figure 5G‐J) and inhibited autophagy (Figure 5K‐N; *P < *0.05) were detected. These findings suggested that the up‐regulation of STK4 could inhibit SW579 cell proliferation while promoting apoptosis and autophagy *via* the Hippo signalling pathway.

### 3.6 Silencing of TNRC6C‐AS1 inhibited tumorigenesis in nude mice

**Figure 6:**
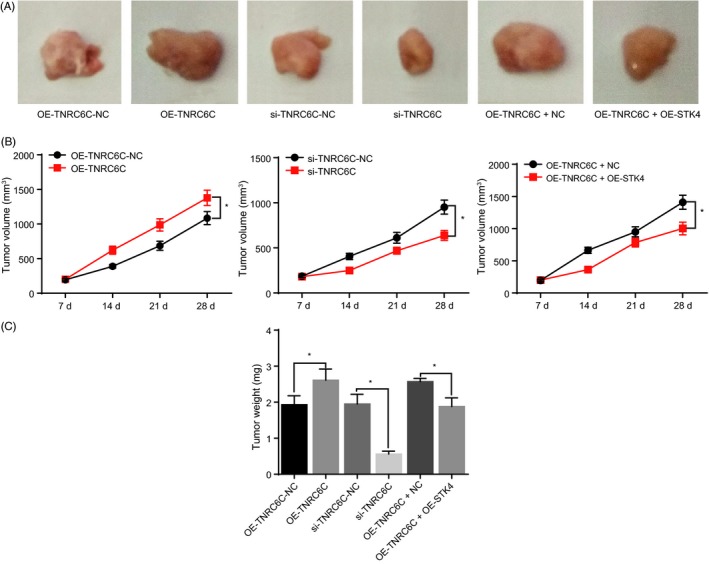
Silencing of TNRC6C‐AS1 inhibited tumorigenesis in nude mice. Panel A, images of resected tumours in each group. Panel B, tumour volumes on 7, 14, 21 and 28 d in each group. Panel C, tumour weights in each group. N = 8; *P < 0.05 vs*. *the OE‐TNRC6C‐NC, si‐TNRC6C‐NC and OE‐STK4 + DMSO groups; the data are analysed using one‐way ANOVA; the experiment was repeated three times

The tumorigenic ability of TC cells was assessed through tumour formation in nude mice. The results (Figure 6A‐C) revealed that the cells in the OE‐TNRC6C group possessed enhanced tumorigenic ability largely due to having larger and heavier tumours than the cells in the OE‐TNRC6C‐NC group (*P < *0.05). A tumour with decreased volume and weight in the si‐TNRC6C group demonstrated its suppressed tumorigenic ability. Compared with the OE‐TNRC6C + NC group, OE‐TNRC6C + OE‐STK4 group exhibited inhibited tumorigenic ability marked by a significant reduction in volume and weight of its tumours (*P < *0.05). The obtained data further demonstrated that the silencing of TNRC6C‐AS1 could inhibit tumorigenesis in nude mice by up‐regulating the expression of SKT4.

## 4 DISCUSSION

According to the *Cancer Statistics in China, 2015*, TC continues to exhibit a substantial upward trend among the female population, particularly those under the age of 30 years old.^20^ Despite the relatively low mortality rate and excellent prognosis, symptoms in approximately 1/10~1/5 patients with differentiated TC may deteriorate either at the early or a recurrent stage of the disease. More recently, treatments aimed at the molecular pathogenesis of TC have been emerged as novel therapeutic targets.^21^ Accumulating evidence has demonstrated the potential role of lncRNAs in TC progression and development. For example, the lncRNA PVT1 has been reported to contribute to the tumorigenesis of TC.^22^ In the present study, we investigated the role of the particular long non‐coding RNA TNRC6C‐AS1 in TC. Our results demonstrated that the silencing of lncRNA TNRC6C‐AS1 could suppress TC cell proliferation and tumorigenesis while promoting apoptosis and autophagy through the inhibition of STK4 methylation *via* the Hippo signalling pathway.

A key initial observation of our study revealed that STK4 was a downstream target gene of lncRNA TNRC6C‐AS1. However, lncRNA TNRC6C‐AS1 could be enriched by DNMT1, DNMT2 and DNMT3 and might regulate STK4 methylation level through binding to CpG island of STK4 promoter. DNA methylation has been defined as an epigenetic mechanism of gene regulation with reports suggesting it may be associated with mammalian development and disease.^23^ A previously conducted functional study asserted that the methylation in CpG island of promoters results in silencing of its downstream genes at a transcriptional level, or the inactivation of tumour suppressor genes in specific cancers.^24^ As a maintenance methyltransferase, DNMT1 is of great significance from a cell survival and tumorigenesis perspective, particularly in the cytosine hypermethylation in CpG islands of tumour suppressor genes.^25^ In addition, reports have suggested that when STK3/4 is inactivated, YAP can translocate to the nucleus and increase the gene expression of its target, which off course was consistent with our results whereby YAP1 translocation from the cytoplasm to the nucleus was enhanced in the si‐STK4 group.^26^ The Hippo signalling pathway has been speculated to result in the phosphorylation of TAZ (transcriptional co‐activator with PDZ‐binding motif), a co‐activator of YAP/transcription. Thus, their respective translocation to the nucleus could be prevented when gene activation is avoided and cell apoptosis is inhibited.^27^

Our study also demonstrated that silencing of lncRNA TNRC6C‐AS1 or the up‐regulation of STK4 could inhibit proliferation and tumorigenicity, which was observed to induce apoptosis and autophagy of the TC cells. Experimental data revealed that, with such interference, the expression of MST1, LAST1, Bax, Beclin‐1 and LC3, as well as the extent of YAP1 phosphorylation, increased while the expression of Bcl‐2 and the activity of YAP1 were decreased. Researchers have demonstrated that STK4 knockout mice exhibit enhanced levels of apoptosis, although STK4 deficiency in humans may elevate the degree of susceptibility to recurrent infections.^26^ More importantly, the loss of STK3/STK4 has been documented to lead to autophagic structure accumulation and increased LC3II expression, which may further affect cells’ ability to fight against infection.^28^ Evidence has been presented suggesting that the LC3 family plays a role in the biogenesis and completion of autophagosomes.^26^ The Hippo signalling pathway plays a crucial role in tissue growth through its downstream effector, YAP1.^29^ Inactivated YAP1 has been speculated to function as a therapeutic target, owing to its ability to reduce tumour volume while inhibiting invasion and metastasis in mice with TC.^30^ Likewise, similar experimental data obtained in a prior study published by Liu et al^27^ revealed that the expression levels of autophagy markers, including Beclin‐1, LC3I and LC3II, were reduced in thyroid papillary carcinoma cells.

## 5 CONCLUSION

**Figure 7:**
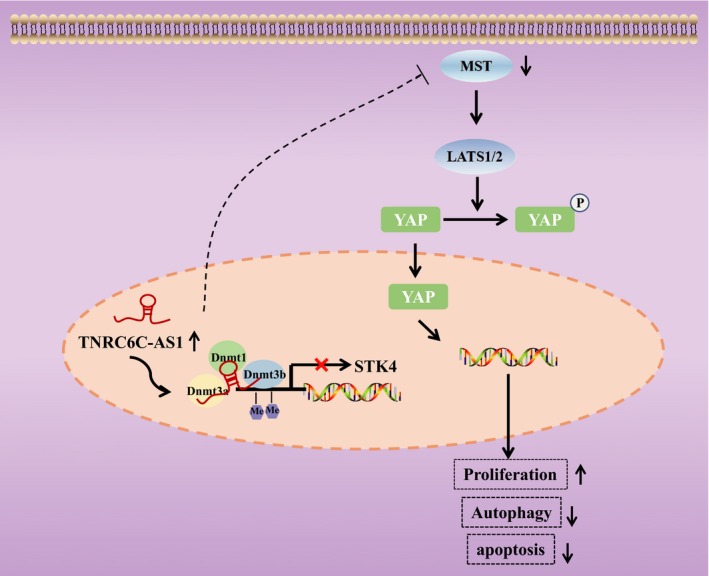
LncRNA TNRC6C‐AS1 facilitated cell proliferation and inhibits cell apoptosis and autophagy by promoting the methylation of STK4 promoter region *via* the Hippo signalling pathway. Over‐expressed TNRC6C‐AS1 suppressed STK4 expression by promoting its methylation through methyltransferase recruitment. The protein levels of MST1 and LATS1/2 as well as the extent of YAP phosphorylation were reduced, thus activating YAP in thyroid carcinoma (TC) cells and promoting its nuclear translocation. As a result, the proliferation of TC cells was promoted while the apoptosis and autophagy of TC cells were inhibited

In conclusion, the key findings presented in our study suggest that the over‐expression of lncRNA TNRC6C‐AS1 is associated with the development of TC through the modulation of cell apoptosis and autophagy by promoting STK4 methylation *via* inhibition of the Hippo signalling pathway (Figure 7). Specific methylation in cancer cells has been highlighted as a biomarker owing to its high sensitivity to aberrant methylation in cancer.^24^ This being said, our study potentially provides a theoretical basis for which lncRNA TNRC6C‐AS1 may be premised upon as a therapeutic target for TC treatment. However, due to the limited sample size and experimental conditions of this study, further studies are required in order to further our understanding in relation to the role of lncRNA TNRC6C‐AS1 in TC.

## CONFLICT OF INTEREST

The authors hereby disclose no conflicts of interest.
